# Optimization of the formula and processing of a sweet potato leaf powder‐based beverage

**DOI:** 10.1002/fsn3.1555

**Published:** 2020-05-07

**Authors:** Dan Luo, Tai‐Hua Mu, Hongnan Sun, Jingwang Chen

**Affiliations:** ^1^ Laboratory of Food Chemistry and Nutrition Science Institute of Food Science and Technology Chinese Academy of Agricultural Sciences Key Laboratory of Agro‐Products Processing Ministry of Agriculture and Rural Affairs Beijing China

**Keywords:** blanching, index of nutritional quality, particle size, suspension stability, sweet potato leaves

## Abstract

For the development of a sweet potato leaf powder (SPLP)‐based beverage, we investigated the effects of blanching methods on SPLP quality (including color, nutritional and functional compositions and antioxidant activity), and the effects of particle size and stabilizers on suspension stability of final product. The total polyphenol and antioxidant activity of SPLP of uncut group were 1.69 and 1.91 times those of cut group, respectively, and the indices of nutritional quality of copper, manganese and vitamin E of uncut group were significantly greater than cut group. The ultrafine SPLP‐produced lowest gravitational sedimentation ratio (49%), indicating it had greatest suspension stability. The optimized formula of SPLP‐based beverage was as follows: ultrafine SPLP of uncut group was mixed with 2.5% (w/w, powder basis) xanthan gum, 1% calcium lactate, 2% ascorbic acid, 12% maltodextrin, 20% xylitol, and 0.9% apple essence. The final product had high nutritional value along with consumer‐acceptable flavor and texture.

## Graphical abstract

Sweet potato leaves are rich in nutrients and functional ingredients. Blanching methods affect the nutritional quality of sweet potato leaf powder differently. Ultrafine powder had a significantly narrower particle size distribution. The final product has a high nutritional value and acceptable sensory qualities.

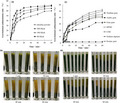

## 1 INTRODUCTION

Sweet potato (*Ipomoea batatas* L.) is a high‐yielding crop belonging to the bindweed or morning glory family, Convolvulaceae. China, the main producer of sweet potato, had an annual production of 72,031,782 tons in 2017 (63.84% of the world total) (FAO, 2018). SPL are the aboveground parts of the sweet potato that can be harvested several times annually. They are an important dark green leafy vegetable in China, having much greater yields than those of other green leafy vegetables (Huang, Chu, Juang, & Wang, 2010). SPL are rich in nutrients and functional ingredients, such as protein, dietary fiber, vitamins, and mineral elements (Sun, Mu, Xi, Zhang, & Chen, 2014). In our previous study, we found that the protein and dietary fiber contents of SPL are 3 and 5 times greater than in the sweet potato roots, respectively. The total polyphenol content (TPC) of SPL from 40 cultivars ranges between 2.73% and 21.39%, and the average polyphenol content is 2 to 3 times greater than that of some commercial vegetables (e.g., kale and spinach). The in vitro antioxidant activities of SPL polyphenols (cultivars Simon No. 1 and Yuzi No. 7) are stronger than those of tea polyphenols, ascorbic acid and grape seed polyphenols (Xi, Mu, & Sun, 2015).

At present, most SPL are discarded as waste in China. Only a fraction of SPL was consumed as a fresh or quick‐frozen vegetable. Owing to the limited processing, storage and transportation opportunities, the nutrient loss from the fresh or quick‐frozen SPL is extremely high. A SPLP‐based beverage product can be prepared by blanching, drying, and grinding SPL. Owing to the low moisture content (less than 10%), SPLP‐based beverages would not only reduce transportation and storage costs, but would also have extended shelf lives. Undesirable browning during the process effects mainly caused by polyphenol oxidase (PPO) and peroxidase (POD) on vegetables greatly affects the sensory appeal and flavor of the final products. Blanching is necessary before drying for which not only inactivates PPO and POD to inhibit the browning effect, but also enhances drying rate and removes pesticide residues (Pandey, Mishra, & Misra, 2019; Xiao et al., 2017). In our previous study, we found that after the SPL were blanched, the PPO and POD activities decreased by 87.64% and 85.7%, respectively (Song, Mu, Sun, & Li‐Sha, 2015). The traditional blanching method often divides the green leafy vegetables (such as mustard, cabbage, and lettuce) into pieces before blanching, resulting in significant color preservation, but the contents of water‐soluble components, such as vitamin C and antioxidant compounds, dropped more than 40% and 50%, respectively (Sengkhamparn, Chanshotikul, Assawajitpukdee, & Khamjae, 2013). In addition, our pre‐experiment revealed that SPLP easily precipitated after mixing with water. The particle size is a key parameter associated with the suspension stability of SPLP. Owing to the lack of an established formula and processing method, the commercialized production of SPLP products is still difficult, which seriously limits the effective development and utilization of SPL.

Therefore, in the present study, the effects of different blanching methods and particle sizes on the quality of SPLP (including color, nutritional and functional composition, antioxidant activity levels, and suspension stability) were investigated. Furthermore, single factor and orthogonal tests were performed to optimize the formula (including stabilizers and flavor agents) of a SPLP‐based beverage. The purpose of this study was to develop a SPLP‐based beverage, with a high nutritional value and acceptable sensory qualities, to improve the effective development and utilization of SPL and further promote the sustainable development of the sweet potato processing industry in China.

## 2 MATERIAL AND METHODS

### 2.1 Plant materials

The fresh SPL from cultivar Simon No. 1 were provided by Haileda Food Co., Ltd., Beijing, China, in the middle of August 2018. After harvesting in the morning, the fresh SPL were immediately sent to our laboratory.

### 2.2 Preparation of SPLP

The SPL were washed with tap water, then divided into the cut (leaves cut into pieces) and uncut (whole leaves) groups, blanched in hot water at 96 ± 2°C for 1 min and then cooled in cold water (15°C). SPL that were cut and not blanched served as the control group, and the three groups were all dried by vacuum freeze dryer. Afterward, the dried SPL were ground and sifted through 80‐mesh, 100‐mesh, and 200‐mesh sieves. The superfine powder was obtained by superfine pulverizer (RT‐04, Beijing Kaichuangtonghe Technology Development Co., Ltd.).

### 2.3 Physical and chemical analysis

#### 2.3.1 Color

Lightness (L*: black = 0 and white = 100, redness and greenness (a*: + = green and − = red), yellowness, and blueness (b*: + = yellow and − = blue) and the total color difference (Δ*E* = $\sqrt{{\left(\Delta L^{*}\right)}^{2}+{\left(\Delta a^{*}\right)}^{2}+{\left(\Delta b^{*}\right)}^{2}}$) value represented the color of the SPLP.

#### 2.3.2 Proximate composition and gross energy

The moisture content was determined using ASAE standards (ASAE, 1983). Crude protein, crude fat, and ash were determined using Association of Analytical Chemists (AOAC) methods (AOAC, 2000). The crude protein content [g/100 g dry weight (DW)] was measured using the micro‐Kjeldahl method, with a nitrogen to protein conversion factor of 6.25 (AOAC method 976.05). The crude fat content (g/100 g DW) was measured using AOAC method 960.39. The ash content (g/100 g DW) was measured by weighing powdered samples before and after a heat treatment (550°C for 12 hr). The crude (g/100 g DW), soluble, and insoluble fiber contents were measured using International Standards Organization (ISO) method 5498 (1981). The carbohydrate content (g/100 g DW) was measured by subtracting the sum of percent ash, crude fat, crude protein, and crude fiber contents from 100. The gross energy (kcal/100 g DW) was measured using a bomb calorimeter with the ISO method 9831 (ISO, 1998).

#### 2.3.3 Mineral content

SPLP was digested in concentrated HNO_3_ (AOAC, 1995). The digest was raised to 25 ml with deionized water. The mineral content was determined by inductively coupled plasma atomic emission spectrometry, expressed in mg minerals/100 g DW.

#### 2.3.4 Vitamin content

The vitamin content was measured as previously reported (Ulusoy & AkAy, 2017; Zhao, Wang, Jiang, & Li, 2017). An aliquot of samples (5 ml) was purified using an Oasis MCX cartridge (6 cc‐150 mg, Waters Corp.) for independent measurements of vitamins C, B1, B2, and B3. High‐performance liquid chromatography‐diode array‐fluorescence analyses were performed using a Waters System (Waters Corp.). The linear gradient elution between solvent A (0.2% H_3_PO_4_ in 20% w/v MeOH) and solvent B (0.2% H_3_PO_4_ in 20% w/v water) was performed by varying B from 100% to 30% from 5 to 10 min. The vitamin contents were expressed as mg of β‐carotene, and the vitamins C, B1, B2, and B3, E and folic acid per 100 g SPLP were expressed on a DW basis.

#### 2.3.5 TPC and antioxidant activity levels

The extraction of crude polyphenols was performed using ultrasound‐assisted ethanol solvent extraction as reported by Sun, Mu, Xi, Zhang, et al. (2014). Briefly, 1 g of sample was extracted with 20 ml of 70% (v/v) ethanol for 30 min at 50°C and subjected to ultrasonic wave treatment. The mixture was centrifuged at 6,000 *g* for 10 min at 4°C, and the residue was re‐extracted two more times as described above. The supernatants were pooled, concentrated, and freeze‐dried to obtain a crude extract. TPC was determined using the Folin–Ciocalteu method with a modification (Sun, Mu, Xi, Zhang, et al., 2014). The quantification was carried out using a calibration curve of chlorogenic acid standards, and the linear regression equation was *y* = 8.7671*x* + 0.0068, *R* ^2^ = 0.9994. The TPC was expressed as chlorogenic acid equivalents (CAE) on a DW basis.

The antioxidant activity of SPLP was determined using the ferric reducing antioxidant power (FRAP) assay described by Lu, Ross, Powers, Aston, and Rasco (2011) with some modifications. The quantification was carried out using a calibration curve of Trolox standards, and the linear regression equation was *y* = 0.0029*x* + 0.017, *R* ^2^ = 0.9918. The antioxidant activity of the SPLP was calculated based on a calibration curve of Trolox and expressed as mg Trolox equivalents (TE) per 100 g on a DW basis.

#### 2.3.6 Amino acid composition

The amino acid compositions of SPLP were measured using a Biochrom 3.1 amino acid analyzer (Amersham) according to a previously reported method (Bártov Aacute, Bárta, Brabcov Aacute, Zdráhal, & Horáčkov Aacute, 2015). The nutritional quality of SPLP was estimated by determining the ratio of essential amino acids and by the amino acid scores (AASs). The amino acid composition of the whole egg, which is considered to be a complete and balanced food, and the adult human's amino acid consumption (FAO/WHO, 2007) pattern were used as references for the estimations of AASs.

#### 2.3.7 Index of nutritional quality

The INQ was calculated according to the following formula (Sun, Mu, Xi, Zhang, et al., 2014):

$$\text{INQ}=\left[\text{Nutrient amount in}\;100\;\text{g DW SPLP}/\text{Chinese nutrient reference value}\;\left(\text{NRV}\right)\right]/\left(\text{Calories in}\;100\;\text{g DW SPLP}/\text{Average energy intake}\right),$$

where the Chinese NRVs for protein, fat, carbohydrate, and fiber are 60 g, ≤60 g, 300 g and 25 g, respectively. The NRVs for calcium (Ca), phosphorus (P), potassium (K), sodium (Na), magnesium (Mg), iron (Fe), zinc (Zn), copper (Cu), manganese (Mn), and selenium (Se) are 800 mg, 700 mg, 2,000 mg, 2,000 mg, 300 mg, 15 mg, 15 mg, 1.5 mg, 3 mg, and 50 μg, respectively. The NRVs for vitamin A, vitamin B1, vitamin B2, vitamin B3, vitamin C, vitamin E, and folic acid are 0.8 mg, 1.4 mg, 1.4 mg, 5 mg, 100 mg, 14 mg, and 0.4 mg. The average energy intake is 2,000 kcal.

#### 2.3.8 Particle size distribution

The particle size distribution was measured from 0.04 μm to 2,000 μm using a laser diffraction particle size analyzer (BT‐9300, Dandong Baite Instrument Co., Ltd) with ultrapure water as the solvent. Briefly, 0.2 g sweet potato leaf powder was mixed with 400 ml ultrapure water, and the particle size was analyzed after circulating for 5 min at room temperature. The particle size distribution was characterized by the volume diameter D[4,3], surface diameter D[3,2], median diameter (D50), and span factor [(D90 − D10)/D50], where D10, D50, and D90 values represent 10%, 50%, and 90% cumulative percentiles of particles (from 0% to 100%) in the distribution.

#### 2.3.9 Suspension stability

Suspension stability was measured using the method of Kosin et al. (2010), with a slight modification. Briefly, SPLP was dispersed in boiled water (60°C) at a powder:water ratio of 1:50 (w/w) and mixed to form a suspended mixture. The suspension stability of SPLP was measured using the gravitational sediment ratio, which was calculated according to the following formula:

$$\text{Gravitational sediment ratio}\;\left(\%\right)=\left(\text{the volume of supernatant}/\text{total volume of suspension mixture}\right)\times 100.$$

### 2.4 Formula optimization

To further improve the dispersion, as well as the sensory properties of SPLP, several downstream processes to prepare a SPLP‐based beverage were investigated, including the addition of stabilizers and flavor agents.

#### 2.4.1 Stabilizers

Seven commonly used stabilizers, xanthan gum, arabic gum, guar gum, hydroxypropyl methylcellulose, carboxymethylcellulose sodium, sodium alginate, and konjac gum were investigated. Briefly, the stabilizers were added individually to the powder–water mixture that was mentioned above at concentrations of 2.5% (w/w), which was determined as the optimal concentration in a preliminary experiment (data not shown).

#### 2.4.2 Flavor agents

Five commonly used flavor agents, xylitol, ascorbic acid, maltodextrin, calcium lactate, and apple essence were investigated according to the formulae of marketed powder‐based beverages. In accordance with the Chinese National Standards, the literature, xylitol, ascorbic acid, maltodextrin, calcium lactate, and apple essence were added to the powder–water mixture to reach concentrations of 0%–40%, 0%–8%, 4%–12%, 0.4%–1.2%, and 0.3%–1.5% (w/w), respectively.

#### 2.4.3 Orthogonal test

Based on the results of the single factor preliminary experimental analysis (date not shown), the additions of xylitol, ascorbic acid, apple essence, and maltodextrin greatly influenced the color, flavor, and other sensory properties of the SPLP‐based beverage, while the addition of calcium lactate had little influence. Therefore, an orthogonal test with four factors and three levels was designed to optimize the formula for the SPLP‐based beverage, including the additions of xylitol, ascorbic acid, apple essence, and maltodextrin. The addition of calcium lactate was determined to be 1% according to the Chinese National Standard GB/T 2760 (2014) for food additives. The parameters of the L_9_(3^4^) orthogonal test are shown in Table 1, and the sensory evaluation standard of the SPLP‐based beverage was the response value.

**TABLE 1:** Factors and levels for the formulation test (% w/w, powder basis)

Factors	A	B	C	D
	Xylitol	Ascorbic acid	Apple essence	Maltodextrin
1	10	2	0.6	8
2	20	4	0.9	10
3	30	6	1.2	12

A panel, consisting of 15 trained members, was used to evaluate the color, smell, texture, and taste of SPLP‐based beverages brewed with boiled water (60°C). Details of sensory evaluation are shown in Table S1. The evaluation was carried out in a descriptive manner using scores for each attribute. The total score was 100, and the average score was used to evaluate the sensory appeal of the product after removing the highest and lowest scores.

### 2.5 Statistical analyses

All the experiments were performed in triplicate. Statistical analyses were performed using the SAS version 9.4 software (SAS Institute Inc., Cary, NC, USA). Statistical significance was set to *p* < .05.

## 3 RESULTS AND DISCUSSION

### 3.1 Color

The color characteristics are listed in Table 2, compared with the control group, whose chromatic L* value was 43.25 ± 0.045, the blanched groups (cut and uncut groups) showed decreasing L* values, resulting in a darker appearance. Similar color changes were reported by Latorre, de Escalada Plá, Rojas, and Gerschenson (2013), in which the changes were correlated with the pigment leakage as consequence of cell membrane thermal denaturalization. In addition, the a* and b* values of the blanched groups were significantly lower than those of the control group, indicating that the blanched groups showed less greenness (lower a*) and less yellowness (lower b*) than the control group, which was probably owing to the loss of water‐soluble components during the blanching. The total difference in color (Δ*E*) indicates the magnitude of the color difference between processed and unprocessed samples. Table 2 shows that the ΔE of cut group (54.16 ± 0.30) and uncut group (54.38 ± 0.59) is slightly higher than that of control group (52.50 ± 0.41). However, Δ*E* differences less than 3 are considered similar in color because the human eye cannot detect any difference (García, Narváez, Heredia, Orjuela, & Osorio, 2017). A previous study reported that residual enzyme activity, the binding state and internal component contents of the material varies depending on the blanching methods (Lopriore et al., 2001). The SPLP made from the blanched groups maintained its color for a long time (>5 hr) after brewing, indicating that the blanching operation had a protective effect on the green coloration.

**TABLE 2:** The effect of different blanching method on the color, proximate composition and gross energy, mineral, vitamin, total polyphenol content (TPC), and antioxidant activity of sweet potato leaf powder

Treatment	Control group	Cut group	Uncut group
Color			
L*	43.25 ± 0.45a	41.14 ± 0.34b	40.83 ± 0.63b
a*	−10.20 ± 0.13a	−8.66 ± 0.12b	−8.33 ± 0.16b
b*	17.50 ± 0.18a	15.42 ± 0.21b	14.83 ± 0.24c
ΔE*	52.50 ± 0.41b	54.16 ± 0.30a	54.38 ± 0.59a
Proximate composition and gross energy (g/100 g DW)			
Moisture^a^	6.22 ± 0.00b	6.66 ± 0.00a	5.74 ± 0.04c
Crude protein	30.05 ± 0.15a	29.55 ± 0.15a	29.85 ± 0.25a
Crude fat	4.12 ± 0.01a	3.96 ± 0.01c	4.04 ± 0.02b
Carbohydrate	14.30 ± 0.00b	9.20 ± 0.00c	17.50 ± 0.00a
Crude fiber	35.00 ± 0.00b	40.40 ± 0.00a	32.80 ± 0.00c
Soluble fiber	7.26 ± 0.10c	9.69 ± 0.10a	8.78 ± 0.01b
Insoluble fiber	27.68 ± 0.16b	30.73 ± 0.16a	24.05 ± 0.09c
Ash	10.41 ± 0.01a	10.08 ± 0.01c	10.18 ± 0.01b
Gross energy^b^	1,184.00 ± 0.00b	1,131.00 ± 0.00c	1,214.00 ± 0.00a
Mineral (mg/100 g DW)			
Na	47.36 ± 0.01c	101.35 ± 0.01a	90.11 ± 2.89b
P	384.10 ± 3.00a	387.40 ± 3.00a	378.00 ± 2.80a
Ca	916.30 ± 4.90b	994.95 ± 4.90a	911.70 ± 5.70b
K	4,803.50 ± 20.50a	2,851.50 ± 20.50b	2,543.50 ± 10.50c
Mg	258.40 ± 0.70a	194.15 ± 0.70c	215.75 ± 8.05b
Fe	12.54 ± 0.10a	10.08 ± 0.10b	8.33 ± 0.39c
Zn	2.65 ± 0.01a	1.71 ± 0.01c	2.06 ± 0.07b
Cu	1.19 ± 0.00a	1.05 ± 0.00b	1.20 ± 0.02a
Mn	16.95 ± 0.04a	8.98 ± 0.04c	10.59 ± 0.36b
Se^c^	5.37 ± 0.15b	5.16 ± 0.15b	6.52 ± 0.30a
Pb^d^	N.D. (<0.04)	N.D. (<0.04)	N.D. (<0.04)
As	0.16 ± 0.01b	0.23 ± 0.01a	0.21 ± 0.00a
Hg^d^	N.D. ( <0.010)	N.D. (<0.010)	N.D. (<0.010)
Vitamin (mg/100 g DW)			
Vitamin C	77.69 ± 0.96a	75.64 ± 0.96a	70.32 ± 0.08b
Vitamin B1^d^	N.D. (<0.12)	N.D. (<0.12)	N.D. (<0.12)
Vitamin B2	1.02 ± 0.01b	1.03 ± 0.01b	1.11 ± 0.00a
Vitamin B3	0.55 ± 0.00a	0.56 ± 0.00a	0.57 ± 0.02a
β‐carotene	82.10 ± 1.60c	126.50 ± 1.60a	121.5 ± 5.00b
Vitamin E	8.00 ± 0.16c	14.70 ± 0.16b	18.90 ± 0.30a
Folic acid^c^	56.43 ± 0.66a	54.03 ± 0.66a	56.36 ± 0.82a
TPC (g CAE/100 g DW)	6.42 ± 0.05A	2.90 ± 0.10B	4.91 ± 0.20C
Antioxidant activity (g TE/100 g DW)	14.76 ± 0.09a	6.10 ± 0.08c	11.66 ± 0.09b

Note

Data are means ± *SD* (*n* ≥ 2). Values within rows with different letters are significantly different (*p* < .05).

^a^ Moisture content was expressed in g/100 g DW.

^b^ Gross energy was expressed in kJ/100 g DW.

^c^ Se content and Folic acid content were expressed in μg/100 g DW.

^d^ N.D. means the content of minerals and vitamin B1 were not detected in sweet potato leaves powder.

### 3.2 The nutrient components of SPLP obtained by different blanching methods

#### 3.2.1 Proximate composition

As shown in Table 2, the moisture contents ranged from 5.74 ± 0.04 g/100 g powder basis (PB) to 6.66 ± 0.00 g/100 g PB. The SPLP made from the cut group had the lowest moisture content (5.74 ± 0.04 g/100 g PB), which could not only reduce the costs of transportation and storage, but also extend the shelf life. Blanching could dissolve the hydrophobic waxy layer and weaken cell walls and membranes. However, the degree of increasing skin permeability was depended on the blanching method, which resulted in the different moisture content in cut group and uncut group (Giovanelli, Brambilla, Rizzolo, & Sinelli, 2012). Furthermore, Wennberg, Ekvall, Olsson, and Nyman (2006) considered the reason attributed the difference was related to the content of crude fiber content in different blanching treatment group.

There was no significant difference between the crude protein contents of the control and blanched groups, indicating that the blanching operation did not cause a loss of protein. Furthermore, the average crude protein content in the SPLP (29.81 g/100 g DW) was 1.64 to 6.62 times greater than that of commercial green tea powder (18.10 g/100 g DW) and commercial barley leaf powder (4.50 g/100 g DW).

The crude fat content of the control group was 4.12 ± 0.01 g/100 g DW, which was greater than that of the cut group (3.96 ± 0.01 g/100 g DW) and that of the uncut group (4.04 ± 0.02 g/100 g DW). Our results were similar to those reported by Sun, Mu, Xi, and Song (2014), in which boiling decreased the crude fat content of SPL significantly. During the blanching process, the volatiles and water‐soluble fatty acids in the SPLP may be partially lost or destroyed, causing a decrease in the crude fat content, and the lowest crude fat content of cut group could be attributed to a higher degree of leaching during the blanching step. Additionally, the use of blanched SPLP with low fat contents as raw materials for our product is in accordance with low‐fat diets.

The carbohydrate content was the highest in the uncut group (17.50 ± 0.00 g/100 g DW), which was significantly greater than that of the control and the cut groups. This result was different from that obtained by Mepba, Eboh, and Banigo (2016), in which blanching had no influence on the carbohydrate contents of some edible Nigerian leafy vegetables. This might be attributed to the different blanching methods and vegetable cultivars. Furthermore, the loss of carbohydrate of cut group resulted from the decreased of the content of water‐soluble low‐molecular weight component such as glucose, fructose, and sucrose (Wennberg et al., 2006). In addition, carbohydrates are the main component and energy‐yielding nutrient for the human body, and their functions include economizing protein, antiketogenesis, and enhancing the energy supply (Jiang, Che, Qin, Kong, & Farouk, 2017).

The average crude fiber content of SPLP was 36.06 g/100 g DW, which was similar to that of commercial tea powder (40.27 g/100 g DW) and that of commercial barley leaf powder (41.33 g/100 g DW). The average soluble and insoluble fiber contents were 8.58 g/100 g DW and 27.48 g/100 g DW, respectively, indicating that SPLP is rich in insoluble fiber. Dietary fiber, especially insoluble dietary fiber, is good for controlling weight and bowel detoxification owing to its high water‐binding and swelling capacities (Tan, Wei, Zhao, Xu, & Peng, 2017).

The ash contents of SPLP ranged from 10.08 ± 0.01 g/100 g to 10.41 ± 0.01 g/100 g DW and that of the blanched group was lower than that of the control group, indicating that blanching decreased the ash content significantly. Our results were similar to those reported by Zoro, Zoué, Adom, and Niamké (2016), who investigated the nutritional and antioxidative properties of blanched leafy vegetables and revealed that blanching had a negative impact on the ash content. This may result from the large amount of cytochylema and that some of the water‐soluble nutrients could be lost during blanching process because the SPL are exposed to water, especially the lowest ash content of cut group.

The gross energy of the uncut group was 1,214.00 ± 00 kJ/100 g DW, which was greater than those of control group (1,184.00 ± 00 kJ/100 g DW) and cut group (1,131.00 ± 0.00 kJ/100 g DW). This could result from the increased carbohydrate level in the uncut group, which is the main energy producing substance.

#### 3.2.2 Minerals

Minerals are classified into two groups: macroelements (Na, P, K, Ca, and Mg) and microelements (Fe, Zn, Cu, Mn, Se, Pb, As, and Hg). As shown in Table 2, the Na content of the control group was 47.36 ± 0.01 mg/100 g DW, and those of the cut and uncut groups increased by 2.14 and 1.90 times, respectively. The average Na content (76.61 mg/100 g DW) was greater than those of kale (43.7 mg/100 g DW) and lettuce (36.5 mg/100 g DW) (Abbey, Pham, Annan, Leke‐Aladekoba, & Thomas, 2018). There was no significant difference between the control and blanched groups in P content, and the average P content was 383.17 mg/100 g DW, which is greater than that of eggplant (311.69 mg/100 g DW) (Nergiz, Selman, Anne, Amy, & Sami, 2018). The Ca content of the cut group (994.95 ± 4.90 mg/100 g DW) was greater than that of the control group and that of the uncut group. In addition, blanching caused large losses in K and Mg. The K content of the control group was 4,803.50 ± 20.50 mg/100 g DW, while those of the cut and uncut groups were reduced by 40.6% and 47.1%, respectively. The Mg content of the control group was 258.40 ± 0.70 mg/100 g DW, while those of the cut and uncut groups were reduced by 24.86% and 16.5%, respectively. Our results were similar to those of Mukherjee and Chattopadhyay (2007), in which the loss of nutrients during hot water blanching was caused mainly by leaching or diffusion. However, the K/Na ratios determined in the cut (28.14) and uncut (28.23) groups were greater than in spinach (18.10) and water‐spinach (11.56) (Taira, Taira, Ohmine, & Nagata, 2013). A low K/Na ratio is correlated with diabetes, high blood pressure and hypokalemia; therefore, SPL powder may be a healthy option for individuals with these conditions.

As for some microelements that are benefit to health, the Fe content of the control group was 12.54 ± 0.10 mg/100 g DW, while those of the cut and uncut groups were reduced by 19.62% and 33.57%, respectively. Zn is an important functional metal in the human body, which has over 300 Zn‐containing enzymes. The average Zn content of SPLP was 2.14 mg/100 g DW, which was 8.03 times greater than in commercial tea powder (0.33 ± 0.00 mg/100 g DW). Cu is an essential dietary microelement that is a cofactor of many redox enzymes and works on many biological processes, including antioxidant defense, neuropeptide synthesis, and immune function. The average Cu content of SPLP was 1.15 mg/100 g DW. The most abundant microelement was Mn, with an average content of 12.17 mg/100 g DW. In addition, Se is an essential antioxidant mineral that performs many functions in the body, including the preservation of normal liver functions and maintaining resistance to disease, and the average Se content of SPLP was 5.68 μg/100 g DW, which was greater than that of cabbage (3.63 μg/100 g DW) but similar to the content of broccoli (Sirichakwal, Puwastien, Polngam, & Kongkachuichai, 2005). However, the minerals content is considered to be a result of the loss of water‐soluble minerals and interaction among other compositions, which resulted in the different minerals content between cut group and uncut group (Ngobese & Workneh, 2018).

There are some microelements that are harmful and toxic, such as Pb, As, and Hg. Neither Pb nor Hg was detected in any of the SPLP, and as content was lower than the limit (0.5 mg/100 g DW) of Chinese National Standard GB/T 29602 (2013) for solid beverages. Thus, SPLP is a safe food product.

#### 3.2.3 Vitamins

SPL are a good source of various vitamins. As shown in Table 2, the most abundant vitamin was β‐carotene, which had an average content of 110.03 mg/100 g DW, which was 32.69 times greater than that of spinach. In addition, the β‐carotene content of the cut (126.50 ± 1.60 mg/100 g DW) and uncut (121.5 ± 5.00 mg/100 g DW) groups was greater than that of the control group, indicating that blanching increased the β‐carotene significantly. This could be attributed to β‐carotene being a fat‐soluble compound, while water‐soluble compounds are lost during the blanching process (Kourouma, Mu, Zhang, & Sun, 2019). Similarly, the vitamin E content of the uncut group was 18.90 ± 0.30 mg/100 g DW, which was 2.36 times greater than that of the control group. The average vitamin C content of SPLP was 74.55 mg/100 g DW, which was greater than those of cucumber (10.60 mg/100 g DW), white radish (22.50 mg/100 g DW), and spinach (39.00 mg/100 g DW) (Isabelle et al., 2010). Vitamin B is an essential nutrient for health, growth, and reproduction because it stimulates metabolism and promotes the conversion of proteins, fats, and carbohydrates into energy. The average vitamin B2 and B3 contents of SPLP were 1.05 mg/100 g DW and 0.56 mg/100 g DW, respectively, which was greater than those of wheat (0.11 mg/100 g DW and 0.16 mg/100 g DW, respectively) (Shewry et al., 2011). There was no significant difference between the folic contents of the control and blanched groups, and the average folic content was 55.61 mg/100 g DW. Interestingly, the retention of vitamin C of cut group was higher than that of uncut group, but the change of fat‐soluble vitamins retention was the opposite. The vitamin C loss in the uncut group probably was related to the mass transfer phenomena rather than to the leakage of nutrients in the blanching processing, but the fat‐soluble vitamins increase in the uncut group probably due to the leakage of the water‐soluble nutrients (Epameinondas, Eleni, Petros, & Ahrné, 2018).

#### 3.2.4 TPC and antioxidant activity

As shown Table 2, the SPLP of the control group had the greatest TPC (6.42 ± 0.25 g CHAE/100 g DW), which was 2.2 and 1.3 times greater than those of cut and uncut groups. The decrease in the TPC agreed with the findings of Sun, Mu, Xi, and Song (2014), in which the effects of domestic cooking methods on the polyphenol of SPL were investigated. They found that boiling reduced the TPC of SPL by 30%. There was a significant difference between the TPC of the cut (2.90 ± 0.10 g CAE/100 g DW) and uncut (4.91 ± 0.20 g CAE/100 g DW) groups, indicating that different blanching methods had significant effects on the TPC of SPLP. This could result from the cut SPL having a greater area that was exposed to water.

As shown in Table 3, the antioxidant activity level of the control group was 14.76 ± 0.09 g TE/100 g DW, which was greater than those of the cut and uncut groups. Decreases of 20.26% and 56.41% in antioxidant activity level were noted in the uncut and cut groups, respectively, which was in agreement with the TPC loss in SPLP. Sun, Mu, Xi, and Song (2014) found that the antioxidant activity of SPL cooked by boiling decreased by 63.82%, and there was a positive correlation between antioxidant activity and polyphenol content in SPL.

**TABLE 3:** The effect of different blanching method on the amino acid of sweet potato leaf powder (g/100 g, DW)

Treatment	Control group	Cut group	Uncut group
Essential and semi‐essential amino acids (EAA)			
Isoleucine	1.04 ± 0.00b	1.15 ± 0.00a	1.08 ± 0.03b
Histidine	0.58 ± 0.00c	0.60 ± 0.00a	0.59 ± 0.00b
Methionine	0.19 ± 0.01a	0.15 ± 0.01b	0.12 ± 0.01c
Valine	1.31 ± 0.01b	1.43 ± 0.01a	1.38 ± 0.03a
Leucine	2.06 ± 0.00c	2.23 ± 0.00a	2.16 ± 0.02b
Phenylalanine	1.37 ± 0.01b	1.46 ± 0.01a	1.46 ± 0.03a
Threonine	1.15 ± 0.01b	1.22 ± 0.01a	1.21 ± 0.01a
Lysine	1.78 ± 0.00b	1.90 ± 0.00a	1.89 ± 0.01a
Non‐essential amino acids (NEAA)			
Aspartic acid	2.80 ± 0.00b	2.75 ± 0.00c	2.86 ± 0.02a
Serine	1.08 ± 0.01b	1.15 ± 0.01a	1.17 ± 0.01a
Glutamic acid	3.16 ± 0.03a	3.22 ± 0.03a	3.15 ± 0.02a
Proline	1.24 ± 0.01b	1.30 ± 0.01a	1.16 ± 0.03b
Glycine	1.33 ± 0.01b	1.41 ± 0.01a	1.39 ± 0.01b
Alanine	1.46 ± 0.00b	1.53 ± 0.00a	1.50 ± 0.02a
Tyrosine	0.80 ± 0.01b	0.85 ± 0.01c	0.92 ± 0.01a
Arginine	1.38 ± 0.00b	1.49 ± 0.00a	1.48 ± 0.02a
Nutritional quality			
Total amino acids(TAA)	22.69	23.80	23.49
Total essential amino acids (TEAA)	9.46	10.35	9.88
TEAA/TAA (%)	41.70	42.55	42.04
Total acidic amino acids (TAAA)	5.96	5.97	6.01
TAAA/TAA (%)	26.27	23.08	25.59
Total basic amino acids (TBAA)	3.74	3.99	3.96
TBAA/TAA (%)	16.49	16.76	16.86

Note

Data are means ± *SD* (*n* ≥ 2). Values within rows with different letters are significantly different (*p* < .05).

#### 3.2.5 Amino acid composition

As shown in Table 3, the total amino acid contents of the cut and uncut groups, with values of 23.80 ± 0.01 g/100 g DW and 23.49 ± 0.25 g/100 g DW, respectively, were significantly greater than control group value of 22.69 ± 0.01 g/100 g DW. The percentage ratio of EAA to total amino acid ranged from 41.70% (control group) to 42.55% (cut group), and a food with a ratio greater than 36% is considered to be an ideal source of protein (Consultation., 2007). Glutamic acid was the most predominant amino acid in all the SPLP, which was different from the results of Lu et al. (2011), in which the main amino acid of SPL was aspartic acid. Several factors contribute to differences in glutamic and aspartic acid contents, including genotype, maturity, and cultivar. The capacity to satisfy the need for nitrogen and EAAs is primarily evaluated by the nutritional values of proteins (Olaofe, Faleye, Adeniji, & Akinsola, 2009). SPLP contained a high concentration of lysine, with an average content of 1.86 g/100 g DW. Lysine is an EAA and a nutritionally critical amino acid that is lacking in the main cereal crops. The total EAAs of the cut and uncut groups were 10.35 g/100 g DW and 9.88 g/100 g DW, respectively, and these values were greater than those of spinach, fragrant green tea and eucalyptus tea (He et al., 2018). Table 3 also shows the total acidic amino acid content, which was greater than the total basic amino acid content in all the groups, indicating that the proteins in SPLP are mainly acidic in nature.

Furthermore, compared with the amino acid reference patterns an adult diet and whole egg protein (Table S2), the amino acid patterns of the SPLP of the control, cut, and uncut groups were similar, but all the amino acid scores were very low (a score of 100 is considered ideal). Methionine and valine were the first and second most limiting amino acids in all the SPLP. Blanching also increased the valine content, but it did not reach FAO/WHO standards. The increase of amino acid content of blanched groups may have been the result of reduction in non‐enzymatic browning, which avoids condensing between amino groups of amino acids in the protein with sugars. Furthermore, heating process could change the composition of nitrogenous compounds and the side chains of protein‐bound amino acids could react with each other. Those reasons resulted in a change of amino acid content during blanching process (Jaworska, Bernaś, & Mickowska, 2011).

#### 3.2.6 Index of nutritional quality

The relationship between the nutrient content in single foods, meals, and diets and the NRV is measured by INQ. A food with an INQ value of 2–6 is a good source of that specific nutrient, and a food with an INQ value greater than 6 is an excellent source of that specific nutrient (Sun, Mu, Xi, Zhang, et al., 2014). As shown in Table S3, the INQ values of protein, P, Mg, Fe, Cu, vitamin B2, and vitamin C in the SPLP from Simon No. 1 were all between 2 and 6, indicating that it was a good source of those nutrients. The INQ values of dietary fiber, Ca, K, Mn, and β‐carotene were all greater than 8, indicating that SPLP of Simon No. 1 was an excellent source of those nutrients. Interestingly, blanching in cut group can increase the INQ values of dietary fiber, Ca, β‐carotene, and vitamin E and blanching in uncut group can increase the INQ values of β‐carotene and vitamin E. The increase of INQ values had obviously positive correlation with the change of those nutrients content during blanching process.

### 3.3 Particle size distribution

The particle size distribution of SPLP is shown in Table 4. Regarding the D[3,2] values, which represent the mean diameters of particles in proportion to the ratio of the surface area to total volume, the values varied from 12.26 ± 0.21 to 47.95 ± 0.43, with the ultrafine and commercial barley leaf powders having the lowest values. Concerning the D[3,2] values, which represent the diameters of spheres that have the same volume as the particles of the system, the values ranged from 41.03 ± 0.17 to 154.39 ± 0.89, with the ultrafine and commercial barley leaf powders having the lowest values. The particle size of the ultrafine powder ranged from (D(10)) 4.99 ± 0.09 to (D(90)) 86.58 ± 0.41 μm, with a median particle size (D(50)) of 30.22 ± 0.12 μm. The particle size distributions of coarsely ground SPLP 80‐, 100‐ and 200‐Mesh fractions and commercial barley leaf powder were much wider, ranging from (D(10)) 7.16 ± 1.69 to (D(90)) 415.94 ± 3.36 μm, and their median particle sizes (D(50)) were 129.27 ± 0.86, 68.39 ± 0.93, 53.54 ± 0.38 and 32.93 ± 0.90 μm, respectively. In addition, the ultrafine powder had a significantly narrower particle size distribution (span = 2.69) than those of coarsely ground SPLP (3.08 ± 0.41–3.23 ± 1.41).

**TABLE 4:** The particle size distribution of sweet potato leaf powder

Treatment	D[3,2] (μm)	D[4,3] (μm)	D10 (μm)	D50 (μm)	D90 (μm)	Span
80‐Mesh	47.95 ± 0.43a	154.39 ± 0.89a	17.62 ± 0.38e	129.27 ± 0.86a	415.94 ± 3.36a	3.08 ± 0.41b
100‐Mesh	25.02 ± 0.26b	96.68 ± 1.18b	11.58 ± 0.52d	68.39 ± 0.93b	222.71 ± 2.56b	3.09 ± 0.37b
200‐Mesh	21.57 ± 0.24b	76.84 ± 0.65c	8.15 ± 0.38c	53.54 ± 0.38c	176.83 ± 1.66c	3.15 ± 0.24c
Ultrafine powder	16.01 ± 0.12c	41.03 ± 0.17d	4.99 ± 0.09a	30.22 ± 0.12d	86.58 ± 0.41e	2.69 ± 0.16a
Commercial barley leaf powder	12.26 ± 0.21c	48.72 ± 3.23d	7.16 ± 1.69b	32.93 ± 0.90d	113.6 ± 9.19d	3.23 ± 1.41c

Note

Data are means ± *SD* (*n* ≥ 2). Values within columns with different letters are significantly different (*p* < .05).

### 3.4 Suspension stability

**FIGURE 1:**
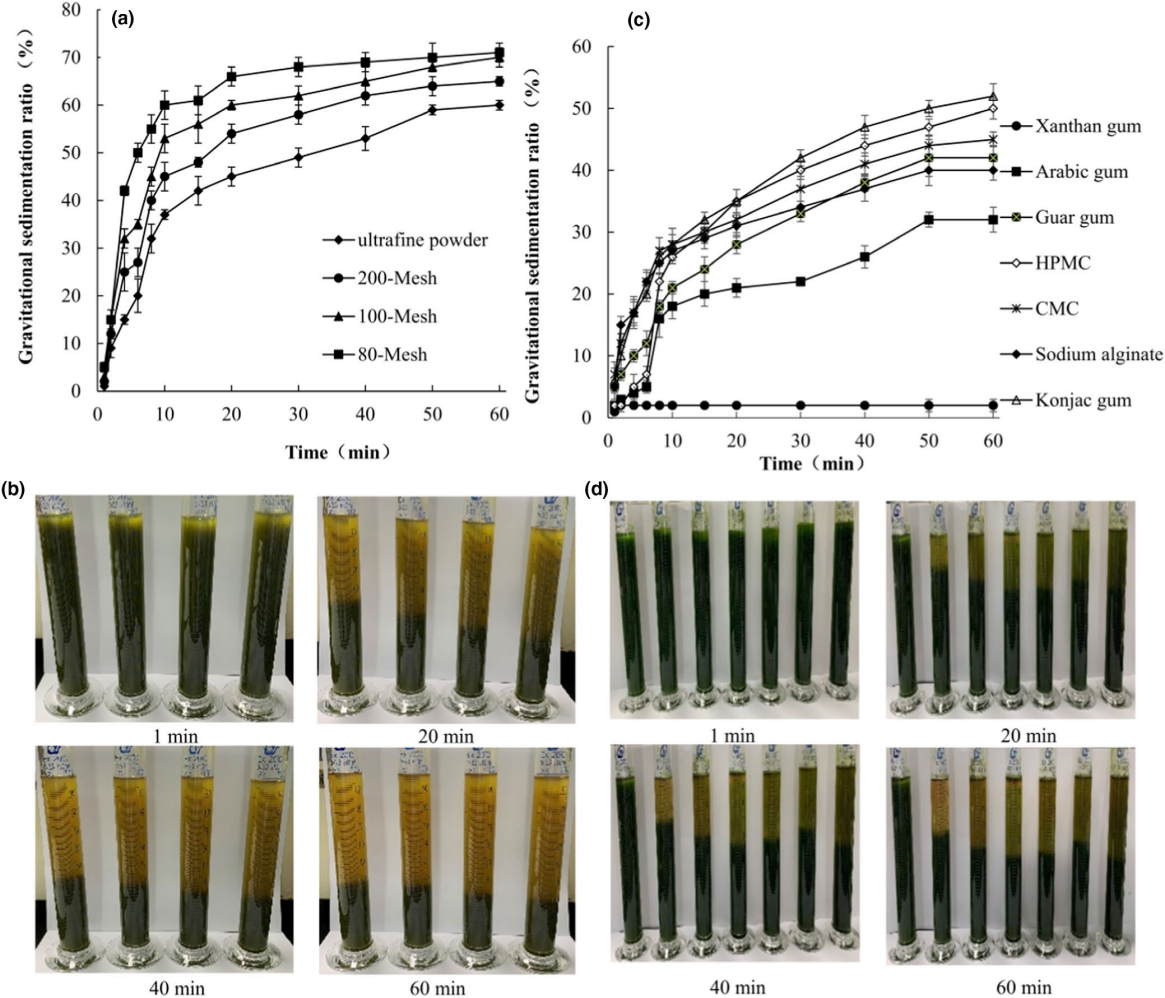
The effect of different particle size and stabilizer on the stability of sweet potato leaf powder suspension. (a) The effect of different particle size on the gravitational sedimentation ratio of sweet potato leaf powder suspension. (b) The effect of different particle size on the sensory phenomenon of sweet potato leaf powder suspension. (c) The effect of different stabilizer on the gravitational sedimentation ratio of sweet potato leaf powder suspension. (d) The effect of different stabilizer on the sensory phenomenon of sweet potato leaf powder suspension. *Note:* In the (b), from left to right were represented ultrafine powder, 200‐Mesh, 100‐Mesh, and 80‐Mesh, respectively. In the (d), from left to right were represented Xanthan gum, Arabic gum, Guar gum, HPMC, CMC, Sodium alginate, and Konjac gum, respectively

Particle size influences the sedimentation behavior and cloudiness of beverages. According to the Stokes' Law and the Derjaguin–Landau–Verwey–Overbeek theory, the smaller the particle sizes and diameters of the solid particles in the suspension system, the more stable the suspension system, because the smaller particle size may result in a slower floating rate (Giri, Mangaraj, Sinha, & Tripathi, 2017). The effects of different particle sizes on the gravitational sedimentation ratio and the sensory phenomenon of SPLP suspension are shown in Figure 1a,b, respectively. In 10 min, the gravitational sedimentation ratio increased sharply from 37% to 60%, as the particle size decreased from 80‐Mesh to ultrafine power. From 10 to 30 min, the gravitational sedimentation ratio increased slightly and the ratio values (in the 30th min) of ultrafine powder, 200‐Mesh, 100‐Mesh, and 80‐Mesh were 49%, 58%, 62%, and 66%, respectively. After standing for 30 min, there was a slight increase in the gravitational sedimentation ratio as particle size decreased. The sensory phenomena of the lower sedimentation layer of all the SPLP suspensions were uniform and delicate. Thus, smaller particles of SPL could be dispersed in water with a higher suspension stability than larger particles. In addition, ultrafine pulverization can improve the digestion rate of food and promote the absorption of nutrients (Field, Duncan, Keller, Stark, & Duizer, 2017).

### 3.5 Formula optimization

#### 3.5.1 Stabilizer

The effects of different stabilizers on the gravitational sedimentation ratio and the sensory phenomena of SPLP suspension are shown in Figure 1c,d, respectively. The smaller the gravitational sedimentation ratio value, the greater the suspension stability. The gravitational sedimentation ratio of the suspended SPLP mixture with xanthan gum was the smallest, and the suspension mixture could be kept uniform and delicate for an hour. The mixture containing arabic gum had a gravitational sedimentation ratio of 32.0%, and the suspension mixture was still uniform and delicate. The effects of guar gum, hydroxypropyl methylcellulose, carboxymethylcellulose sodium, sodium alginate, and konjac gum were less desirable. Additionally, xanthan gum can effectively improve suspension stability of SPLP‐based beverage owing to the ability to create a strong physical network in the system to resist the gravitational force to prevent particles from sinking (Muhammad et al., 2019).

#### 3.5.2 Flavor agents

As shown in Table 5. The order of influence for the various factors on the flavor agents of SPLP‐based beverages was determined using a range analysis as follows: B (ascorbic acid) > D (maltodextrin) > A (xylitol) > C (apple essence). According to the variance analysis (Table S4), there were significant differences in the scores among different levels of each factor. When ascorbic acid was gradually added, the score decreased, while when maltodextrin was gradually added, the score increased. The fourth group had the greatest score (93.1 ± 2.09), indicating that the best formula for flavor agents was 2% ascorbic acid, 12% maltodextrin, 20% xylitol and 0.9% apple essence. The sweet potato leaf powder‐based beverage obtained using this formula had a high nutritional value and desirable flavor and texture.

**TABLE 5:** Orthogonal test result of flavor agent

No.	A (Xylitol)	B (Ascorbic acid)	C (Apple essence)	D (Maltodextrin)	Color	Smell	Texture	Taste	Total
1	1 (10)	1 (2)	1 (0.6)	1 (8)	23.2	14.5	11.5	14.2	63.4 ± 3.80d
2	1	2 (4)	2 (0.9)	2 (10)	22.5	15.3	16.7	21.5	76.0 ± 3.69b
3	1	3 (6)	3 (1.2)	3 (12)	13.2	6.8	13.2	18.9	52.1 ± 2.57e
4	2 (20)	1	2	3	29.6	18.6	18.5	26.4	93.1 ± 2.09a
5	2	2	3	1	25.5	14.2	13.4	14.7	67.8 ± 2.05c
6	2	3	1	2	14.2	16.5	12.1	13.5	56.3 ± 7.19e
7	3 (30)	1	3	2	23.5	15.2	13.7	22.5	74.9 ± 5.67b
8	3	2	1	3	24.2	15.4	11.4	23.5	74.5 ± 2.29b
9	3	3	2	1	5.7	10.3	12.5	8.0	36.5 ± 2.87f
K1	63.8	77.1	64.7	55.9					
K2	72.4	72.8	68.5	69.1					
K3	62.0	48.3	64.9	73.2					
R	10.4	28.8	3.8	17.3					

Note

K was represented the average value of each levels of factors.

R was represented the range value of each factor.

Data are means ± *SD* (*n* ≥ 2). Values within columns with different letters are significantly different (*p* < .05).

## 4 CONCLUSION

The SPLP made from the blanched groups had color‐protective and high nutritional value. In addition, the powder of the uncut group had a greater TPC and antioxidant activity level as compared with those of the cut group. The ultrafine SPLP showed a markedly narrower particle size distribution and high suspension stability. We developed and optimized a formula for a SPLP‐based beverage that further improved its suspension stability and flavor by adding a stabilizer and flavor agents, respectively. The optimized formula was as follows: the ultrafine SPLP of the uncut group was mixed with 2.5% xanthan gum, 1% calcium lactate, 2% ascorbic acid, 12% maltodextrin, 20% xylitol, and 0.9% apple essence. The SPLP‐based beverage obtained by this formula had a high nutritional value and desirable flavor and texture. A SPLP‐based beverage may improve the effective development and utilization of SPL and further promote the development of the sweet potato processing industry in China.

## CONFLICT OF INTEREST

The authors declare no competing financial interest.

## ETHICAL APPROVAL

Ethics approval was not required for this research.

## Supporting information

Table S1‐S4
