# Supplementary material for: Optimization of the formula and processing of a sweet potato leaf powder‐based beverage
Source: Food Sci Nutr. 2020 May 7;8(6):2680–91. doi: 10.1002/fsn3.1555 (PMC7300073; doi:10.1002/fsn3.1555)
Supplement: Supplementary file 1 — Table S1‐S4 [file FSN3-8-2680-s001.docx]

**Table S1 Sensory evaluation standards of sweet potato leaf powder-based beverage**

| Attributes | Proportions | Characteristic | Score |
| --- | --- | --- | --- |
| Color | 30 | Bright green | 25~30 |
|  |  | yellow-green | 15~24 |
|  |  | Brown | 1~14 |
| Smell | 20 | Fresh sweet potato leaves aroma and apple aroma | 14~20 |
|  |  | Apple aroma too strong | 7~13 |
|  |  | Unpleasant | 1~6 |
| Texture | 20 | Rapid dissolution, uniform distribution | 14~20 |
|  |  | Dissolve after stirring, with slight stratification | 7~13 |
|  |  | Insoluble, with impurities or precipitation, layered significantly | 1~6 |
| Taste | 30 | Sweet potato leaves and apples mixed clear fragrance, delicate taste, moderate sweetness | 20~30 |
|  |  | A little sweet potato leaves and apples fragrant flavor, not delicate, no sweetness or too sweet | 10~19 |
|  |  | Tasteless or heavy taste | 1~9 |
| Total | 100 |  |  |

**Table S2 The effect of different blanching method on the amino acid score of sweet potato leaf powder（g/100g DW）**

| Amino Acids | Reference FAO / WHO standard (2007, g/100g) | AAS of control group | AAS of cut group | AAS of uncut group |
| --- | --- | --- | --- | --- |
| Isoleucine | 3.00 | 34.67 | 38.33 | 36.00 |
| Methionine | 2.20 | 8.64 | 6.82 | 5.45 |
| Valine | 3.90 | 33.59 | 36.67 | 35.38 |
| Leucine | 5.90 | 34.92 | 37.80 | 36.61 |
| Phenylalanine + Tyrosine | 3.00 | 72.33 | 77.00 | 79.33 |
| Threonine | 2.30 | 50.00 | 40.67 | 52.61 |
| Histidine | 1.50 | 38.67 | 40.00 | 39.33 |
| Lysine | 4.50 | 39.55 | 42.22 | 42.00 |
| 1 limiting amino acid |  | Methionine | Methionine | Methionine |
|  | Whole egg pattern |  |  |  |
| Isoleucine | 5.40 | 19.26 | 21.30 | 20.00 |
| Methionine | 5.70 | 3.33 | 2.63 | 2.11 |
| Valine | 6.60 | 19.85 | 21.67 | 20.91 |
| Leucine | 6.60 | 31.21 | 33.79 | 32.73 |
| Phenylalanine + Tyrosine | 9.30 | 23.33 | 24.84 | 25.59 |
| Threonine | 4.70 | 24.47 | 25.96 | 25.74 |
| Histidine | 2.20 | 26.36 | 27.27 | 26.82 |
| Lysine | 7.00 | 25.43 | 27.14 | 27.00 |

**Table S3 The effects of different blanching methods on the INQ of sweet potato leaf powder**

| Treatment | Control group | Cut group | Uncut group |
| --- | --- | --- | --- |
| Crude protein | 4 | 4 | 3 |
| Crude fat | ＜1 | ＜1 | ＜1 |
| Carbohydrate | ＜1 | ＜1 | ＜1 |
| Crude fiber | 10 | 12 | 9 |
| Ca | 8 | 9 | 8 |
| P | 4 | 4 | 4 |
| K | 17 | 11 | 9 |
| Na | ＜1 | ＜1 | ＜1 |
| Mg | 6 | 5 | 5 |
| Fe | 6 | 5 | 4 |
| Zn | 1 | 1 | 1 |
| Cu | 6 | 5 | 6 |
| Mn | 40 | 22 | 24 |
| Se | 1 | 1 | 1 |
| Vitamin C | 6 | 6 | 5 |
| Vitamin B1 | ＜1 | ＜1 | ＜1 |
| Vitamin B2 | 5 | 5 | 5 |
| Vitamin B3 | 1 | 1 | 1 |
| Vitamin E | 4 | 8 | 9 |
| β-carotene | 17 | 26 | 25 |
| Folic acid | 1 | 1 | 1 |

A food between 2 and 6 in the INQ ranking system is considered good, and above this is viewed as excellent source.

**Table S4 Variance analysis table**

| Source | DF | Squares Sum of | Mean Square | F Value | Pr > F |
| --- | --- | --- | --- | --- | --- |
| Model | 8 | 17221.94444 | 2152.74306 | 120.66 | <0.0001 |
| A(Xylitol) | 2 | 1488.86111 | 744.43056 | 41.73 | <0.0001 |
| B(Ascorbic acid) | 2 | 11554.19444 | 5777.09722 | 323.81 | <0.0001 |
| C(Apple essence) | 2 | 220.77778 | 110.38889 | 6.19 | 0.0035 |
| D(Maltodextrin) | 2 | 3958.11111 | 1979.05556 | 110.93 | <0.0001 |
| Error | 63 | 1124.00000 | 17.84127 |  |  |
| Corrected Total | 71 | 18345.94444 |  |  |  |
